# Drivers, uncertainties, and future scenarios of the Iranian health system

**DOI:** 10.1186/s12913-022-08774-w

**Published:** 2022-11-23

**Authors:** Mozhgan Emami, Ali Akbar Haghdoost, Vahid Yazdi-Feyzabadi, Mohammad Hossein Mehrolhassani

**Affiliations:** 1 PhD by Research Student in Management Sciences (Health Policy), Health Services Management Research Center, Institute for Futures Studies in Health, Kerman University of Medical Sciences, Kerman, Iran; 2 Epidemiology, Modeling in Health Research Center, Institute for Futures Studies in Health, Kerman University of Medical Sciences, Kerman, Iran; 3 Health Policy, Social Determinants of Health Research Center, Institute for Futures Studies in Health, Kerman University of Medical Sciences, Kerman, Iran; 4 Department of Health Management, Policy and Economics, Faculty of Management and Medical Information Sciences, Kerman University of Medical Sciences, Kerman, Iran; 5 Health Services Management, Medical Informatics Research Center, Institute for Futures Studies in Health, Kerman University of Medical Sciences, Haftbagh Highway, Kerman, Iran

**Keywords:** Health system, Systems analysis, Uncertainty, Drivers, Scenario planning

## Abstract

**Background:**

Health promotion is an essential dimension of sustainable development in any country. It has a high degree of complexity, with numerous components interacting both inside and outside of the system, so having a systemic and forward-looking approach is essential to planning for the future.

**Methods:**

The research has been designed based on scenario-based planning in three main stages. The data gathering was qualitative by working group meetings and compiling an importance-uncertainty questionnaire to complete the cross-impact analysis matrix. The MicMac and scenario Wizard has been used for data analysis.

**Results:**

The scoping review and upstream document evaluation lead to 54 key variables for analyzing the Iranian health system (HS). The MicMac analysis ends by determining seven key variables: power, politics, and communication network; lifestyle and behavioral factors; quality of human resources training and education; environmental and occupational risk factors, payment and tariff system, and allocation pattern; support society / individuals health; and services effectiveness, especially para-clinical and outpatient ones. Finally, six main scenario spaces are depicted using Scenario Wizard. collective equity was the priority of the HS vision in the desirable scenario, consisting of the most favorable state of the uncertainties. The second, third, and fourth scenarios are also considered desirable. In the disaster scenario, which is the most pessimistic type of consistent scenario in this study, health and equity are not significant either in the social or individual dimensions. In the sixth scenario, the individual dimension of health and equity is the most critical perspective of the HS.

**Conclusions:**

Due to the unsustainability and high complexity of the Iran’s HS, the development and excellence of the HS governance based on the Iran context and health advocacy improvement (applying good governance); creating sustainable financial resources and rational consumption; and human resources training and education are three main principles leading the HS to the images of the desired scenarios.

## Background

As a public right, health is one of the most complex issues of community management and development [1]. Therefore, health sector is an essential socio-economy sector that receives a considerable share of public resources, both financial and human [2].

HS’s are paradigmatic examples of human organizations that combine many different professional and disciplinary features in a critical functional environment. The failure of communications and defective processes in HS’s has a tremendous impact on society, both financially and humanly [3] and as a result, is a barrier to achieving better and fairer health outcomes [4]. Traditionally, HS’s have been viewed as linear hierarchical structures. However, recent developments in complexity science point to HS’s as complex entities governed by non-linear interaction laws, self-organization and emergent phenomena [3] and figure out multifaceted and interconnected relationships between components of the HS and the views, interests, and strengths of its various actors and stakeholders. Therefore, system thinking, which has long been used in other fields, has great unused potential, especially in low- and middle-income countries [4].

In other words, over the past two decades, global health stakeholders have realized the importance of dealing with the complex systemic and adaptive nature of HS’s to anticipate better the unexpected and unpredictable consequences of current and new policies. This was made possible by the increasing interest in topics such as systems thinking, complex adaptive systems, and systems science in the published health literature during the past 20 years [5].

According to a recent publication by the World Health Organization, systems thinking can open up powerful avenues for identifying and addressing HS challenges and it is an essential element in strengthening the HS. Thus, WHO has advocated broad approaches such as systems thinking in evaluating the components of the HS, which include service delivery, human resources, information, medical products and technologies, financing, and governance [6].

Systems thinking considers the system as a whole rather than its individual parts, taking into account systems behavior over time as a complex system [7]. It is a way of viewing the world using the general logic underlying various systems theories (such as general systems theory, chaos theory or complexity theory), which is informed by a wide range of related tools and methods (such as system dynamics modeling, structured conceptualization, or network analysis) whose choice depends largely on the question at hand, context, and available capacity [4].

Strategic decision-making processes and forward-looking planning have also been the critical issues discussed by HS stakeholders, besides having a systemic view of health [8]. The future world will be full of changes and instability, and only those who can move towards them proactively can tolerate these changes. The pace of change is so fast that traditional methods cannot help cope with it. Therefore, nations need to be forward-looking to deal with the driving changes affecting all aspects of their lives. Public health foresight and, consequently, health and science foresight are essential in promoting health and solving related problems [1].

The foresight science has facilitated future studies while transforming future-oriented planning studies into science with precise principles and methods. The process of foresight helps to identify and introduce the driving forces, analyze their interconnected direct and indirect effects in a dynamic system and determine the critical variables of planning for the future [9] and finally, provide the necessary facilities for policymakers regarding strategic and long-term decision making [1].

Relatively specific elements and uncertainties of the future can be explained with a set of scenarios, and based on that, take a stance and plan for the future. Scenario analysis is a popular tool for future discovery and plausible planning. Scenario planning is actually a tool used in the decision-making process, and it is very useful when the decision-maker is dealing with issues under uncertainty, that is, a situation where at least one of the decision parameters is not a certain decision [10].

One of the methods of creating a scenario is the method of cross-impact analysis (CIA), which is considered to be one of the soft approaches to systems thinking. Based on the structured approach, statements related to logical relationships between factors and their outcomes are presented. Such statements are usually elicited by asking the respondent to describe which pairs of outcomes are consistent, meaning that these outcomes are likely to occur jointly. Typically, these cross-impact statements are verbalized and then mapped to corresponding numerical parameters. Finally, the extracted propositions are algorithmically combined and provide suggestions that will be combinations of outcome scenarios [11, 12].

In general, it is concluded that systems thinking provides the basis for creating scenarios in a group work and joint thinking, considering the effective interactions between the components of the HS and the context and analyzing the cross-impact between them and carefully examining the possible outcomes of policies and actions [7].

Many studies have examined the interactions among critical variables of the health sector. For example, identifying the interactions and communication networks among critical factors affecting direct out of pocket payments [13], Stankov et al.’s study in 2019 aimed at identifying the factors affecting urban health through a systemic approach and using the cross-impact analysis matrix to understand the power and nature of the component’s relationships and to identify possible future urban health scenarios in Latin America [14], using the cross-impact analysis matrix to develop possible scenarios for hospital supply chain resilience [15], and as well as the use of interaction matrices to examine risk factors for diseases such as cardiovascular disease [16]. However, in the present study, the interaction of the components in all dimensions of the HS has been investigated.

The present study aims to identify the practical components of the HS, the relationships between them, and their degree of influence-dependency using the cross-impact analysis method and providing the images of the HS futures using scenario development.

## Methods

### Study setting

This study is part of a larger research project titled Developing Iran’s HS Scenarios using systems thinking approach: dynamics system modeling, strategic assumption surfacing and testing (SAST). The current study is approved by the Research Ethics Committees of the Kerman University of Medical Sciences (IR.KMU.REC.1397.287) and all methods were performed in accordance with the relevant guidelines and regulations.

The HS in Iran operates in the form of a coherent network, where three levels form the said network, and the movement of patients between levels takes place in the form of a referral system. In Iran, the public and private sectors are responsible for providing various healthcare services together. But mainly the government sector, especially the Ministry of Health and Medical Education (MoHME), has a greater share in this field. The related organizations are listed below: MoHME, private sector, social security organization, charitable organizations, foundations, welfare organization, Ministry of Oil and other government organizations and institutions.

In Iran, the set of financial resources necessary to provide healthcare services is provided through the public sector (government), the social security organization and the private sector (households). A limited percentage of financial resources are also provided in centers affiliated to the non-governmental sector [17].

Many problems are plaguing the health system in Iran, which necessitates a systemic approach to solve them. The main problems are summarized as follows:

1. The problem of economic access of some people to medical services;
2. Lack of information and applied research for policy and planning purposes;
3. Lack of people’s participation in the decision making process;
4. Insufficient inter-departmental coordination;
5. Weak management at some network levels;
6. Defect of referral system;
7. Lack of coordination between the private sector and the public sector
8. Insufficient information system for planning and monitoring
9. Deficiency of social outlook and professional skills in university graduates
10. Economic problems to employ trained personnel
11. Lack of attention to health economics
12. The problem of procurement and supply of some medicines, medical equipment and supplies from abroad
13. The problem of maintenance of existing equipment
14. Lack of supervision in the private sector [18].

### Study sample

This study is practical in purpose and descriptive-analytical using cross-impact analysis. The population’s interest was experts who specialized in health management and policy or had practical systemic-health-related experience, selected purposefully and available. In fact, 10 experts were selected from Isfahan University of Medical Sciences, Shiraz University of Medical Sciences, Institute for Futures studies in Health affiliated to Kerman University of Medical Sciences and Ministry of Health Strategic Planning.

### Research methods

Data collection and analysis have been done in three stages using cross-impact research and scenario development. In the first step, a list of critical variables was created as the output of a scoping review [19] and reviewing upstream documents. Regarding the review of documents, the following reports were also investigated:

1. The economic status of the Iran’s HS;
2. Monitoring the status of medical services in the Iran’s HS;
3. Monitoring the status of providing rehabilitation services in the Iran’s HS;
4. Monitoring the status of palliative services in the Iran’s HS;
5. Descriptive epidemiology of risk factors and risky behaviors in Iran;
6. The status of demographic indicators and its structure by provinces of the country;
7. Compilation of HS manpower indicators;
8. World Health Organization report;
9. Development plan;
10. Descriptive epidemiology of disease burden in Iran;
11. Health financing model in Iran;
12. Monitoring of primary health care provision in Iran;
13. Monitoring the knowledge management status in the Iran’s HS;

The identified variables were provided to experts in a scoring questionnaire in the second stage. Items such as “data reliability,” “quantification capability,” “data access rate,” and “agent clarity rate” were asked to be scored, which helps to select and prioritize essential components, based on the Likert score 1–5 (1 = very low, 2 = low, 3 = medium, 4 = high and 5 = very high) and the items of “importance” and “uncertainty” were scored with a range of 1–10 by experts to investigate component’s influence on achieving the health of the Iranian society in future and their uncertainty degree.

In parallel with completing the questionnaire, a matrix was designed in Excel consisting of the 54 key variables in the form of a 54 * 54 that shows the effects of the variables in rows on those in columns. It was completed in repeated sessions and two rounds by experts based on the rate of the effectiveness of each variable between 0 to 3. The number one stands for weak effects, two for moderate effects, three for substantial results, and 0 indicates no effect. This matrix is called the direct effects matrix.

In the next step, the cross-impact matrix was entered into MicMac software. In addition to plotting direct effects between variables, indirect effects can also be identified in MicMac. It will provide an algorithm for locating variables on direct and indirect effects maps. According to this map, variables are also classified into four quadrants/zones. Zone one belongs to strategic or two-dimensional variables that are both effective and influential and are located in the northeastern part of the map. They are both manipulable and controllable and affect system dynamics and change, forming indicators of instability. The variables above the diagonal line of this area are called risk variables, and the variables below the diagonal line are called the target variables and display the system results. The variables in Zone two, shown in the northwestern part of the map, are considered the most critical components, more effective and less influential, and are considered input variables. The variables of zone three have low impact and influence in the southeastern part of the map and are called independent and exceptional variables. There are three types of variables in this section as follow:

1. Discrete variables (located near the origin of the coordinates and not related to current system dynamics and changes);
2. Secondary lever variables (although entirely independent, they are more effective than being affected, located above the diagonal line, and can be used as benchmarks or measurement points);
3. Regulatory variables (located below the diagonal line and close to the center of the origin of the coordinates. Can be analyzed as secondary leverage, weak targets, and secondary risk variables).

Finally, the variables of Zone four are not strategic due to their dependency that other types of variables are influencing them.

**Fig. 1:**
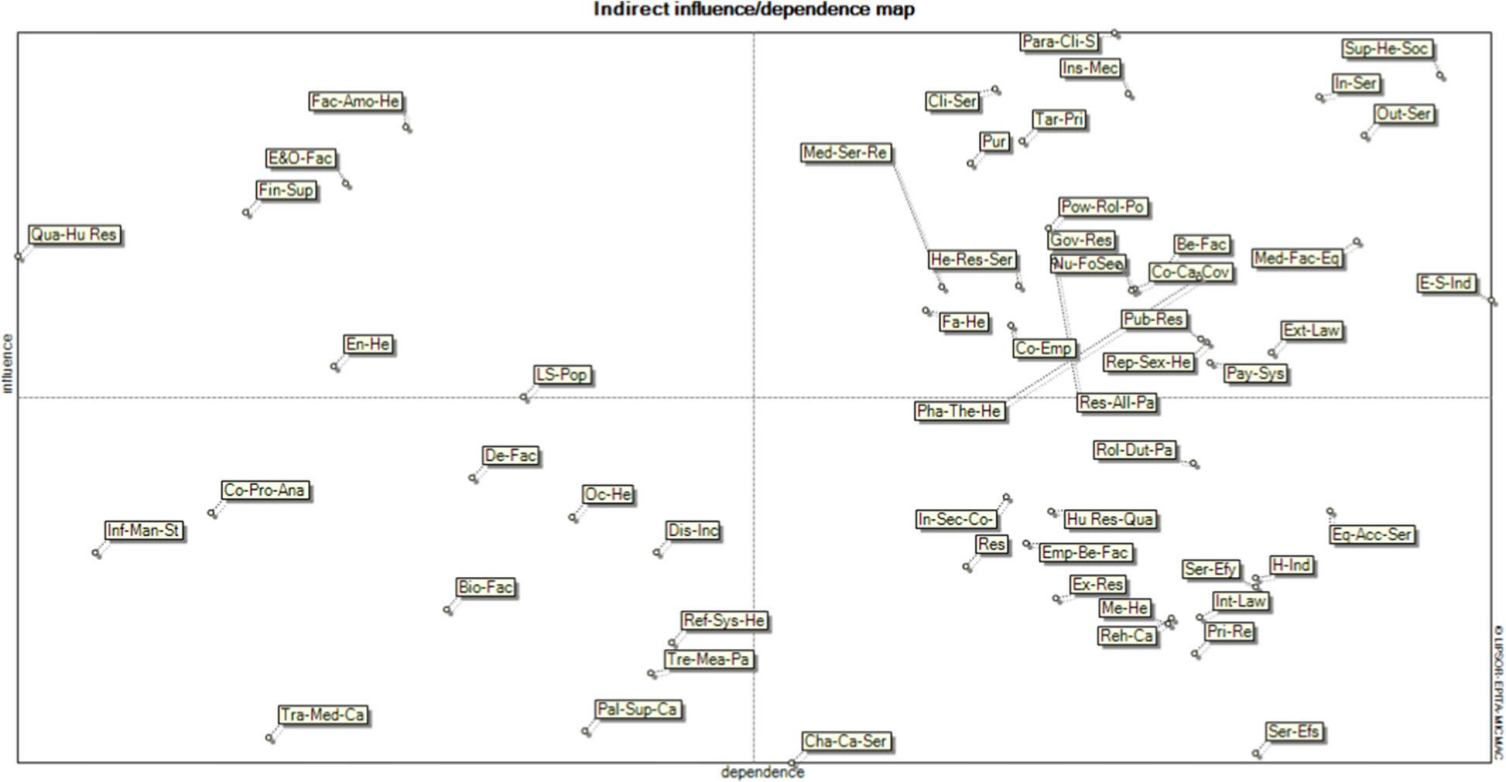
Results of Analysis of Indirect Effects of Variables in the Iranian HS

In the third stage, the variables that had the most importance and uncertainty (degree of importance> 7 and uncertainty>, five or close to 5) (Fig. 3) and located in the upper left quarter and above the bisector of the lower-left quarter (as secondary levers) of the MicMac map (Fig. 1) were identified and categorized as the main drivers and entered in Scenario Wizard software.

The Scenarios framework was determined in working group meetings based on previous phases’ driving forces and uncertainties analysis and for each of them, alternatives were set. Then, the effect of each of the uncertainties/drivers alternatives was measured relative to each other using scenario wizard, and based on statistical calculations, compatible future scenarios were determined. Accordingly, the matrix of cross-effects was scored by health experts with the numbers − 3 to + 3. Number − 3 indicates the severe or strong limiter effect, − 2 indicates moderate limiter, − 1 indicates weak limiter, 0 stands for ineffectiveness, + 1 indicates weak amplifier, + 2 shows moderate amplifier, and + 3 stands for a robust amplifier. Finally, consistent scenarios were developed using the final score of the participants, and the outputs’ validity was assessed by referring to the opinions of experts. In the scenario narration phase, a systematic analysis of the drivers and the key variables was done in each scenario space using the opinions and qualitative views of researchers in a working group meeting.

## Results

Based on the scoping review and review of upstream documents, 54 key variables were identified to analyze the Iranian HS (Table 1). Then, after obtaining and summarizing experts’ opinions in completing the cross-impact matrix, the results were drawn in MicMac software in the form of tables, charts, and maps of direct and indirect effects.

Table 1 shows the main variables of the system, their influence, and dependency in form of figures and the location of the variable on the map. It should be noted that the numbers for each variable in the row show the influence and, in the column, the degree of dependency.

**Table 1:** Results of analysis of direct effects of variables in the Iranian HS

N°	D**imensions of the** H**eath system**	C**omponents**	V**ariables**	T**otal number of rows**	T**otal number of columns**	L**ocation of variables in the direct effects analysis map**
1	**Population health**	**Demographics**	Demographic factors related to population/patient (De-Fac)	107	100	Lower-left area of the map (third quarter-secondary leverage)
2			Population lifestyle change (LS-Pop)	113	102	On the horizontal axis (influence axis- secondary leverage)
3		**High-risk behaviors and risk factors**	Behavioral risk factors (Be-Fac)	122	119	Upper- right area of the map (first quarter-target variable)
4			Biological risk factors (Bio-Fac)	97	100	Lower-left area of the map (third quarter-adjustable variable)
5			Environmental and occupational risk factors (E&O-Fac)	128	97	Upper-left area of the map (Second quarter-drivers)
6		**Community health status**	Health indicators (H-Ind)	100	121	Lower-right area of the map (fourth quarter-dependent variable)
7			Economic and social indicators (E-S-Ind)	119	127	Upper-right area of the map (first quarter-target variable)
8			Disease incidence (Dis-Inc)	102	105	Lower-left area of the map (third quarter-adjustable variable)
9	**Service delivery**	**Preventive services**	Community empowerment (Co-Emp)	118	115	Upper-right area of the map (first quarter-target variable)
10			**Community care covered (Co-Ca-Cov)**	121	118	Upper-right area of the map (first quarter-risk variable)
11			Environmental health (En-He)	115	97	Upper-left area of the map (second quarter-drivers)
12			Mental health (Me-He)	96	119	Lower-right area of the map (fourth quarter-dependent variable)
13			Nutrition and food security (Nu-Fo Sec)	120	118	Upper-right area of the map (first quarter-target variable)
14			Occupational health (Oc-He)	104	103	Lower-left area of the map (third quarter-adjustable variable)
15			Family health (Fa-He)	118	112	Upper-right area of the map (first quarter-risk variable)
16			Reproductive and sexual health (Rep-Sex-He)	116	120	Upper-right area of the map (first quarter-target variable)
17		**Medical service**	**Outpatient services delivery (Out-Ser)**	133	123	Upper-right area of the map (first quarter-target variable)
18			Para-clinical services (Para-Cli-Ser)	139	117	Upper-right area of the map (first quarter-risk variable)
19			Clinical services (Cli-Ser)	135	114	Upper-right area of the map (first quarter-risk variable)
20			Inpatient services delivery (In-Ser)	135	122	Upper-right area of the map (first quarter-risk variable)
21		Palliative and supportive care coverage (Pal-Sup-Ca)		89	103	Lower-left area of the map (third quarter-adjustable variable)
22		Rehabilitation care coverage (Reh-Ca)		97	119	Lower-right area of the map (fourth quarter-dependent variable)
23		Traditional medical care coverage (Tra-Med-Ca)		88	95	Lower-left area of the map (third quarter-adjustable variable)
24		**Improving services quality**	Treatment measures/patient rights (Tre-Mea-Pa-Rig)	93	105	Lower-left area of the map (third quarter-adjustable variable)
25			Equity and access to services (Eq-Acc-Ser)	105	123	Lower-right area of the map (fourth quarter-dependent variable)
26			Services effectiveness (Ser-Efs)	87	121	Lower-right area of the map (fourth quarter-dependent variable)
27			Services efficiency (Ser-Efy)	98	121	Lower-right area of the map (fourth quarter-dependent variable)
28		**Leveling and service delivery levels**	Improvement of health referral system (Ref-Sys-He)	95	106	Lower-left area of the map (third quarter-adjustable variable)
29			Characteristics and capacity defined for each level of service (Cha-Ca-Ser)	87	109	Lower-right area of the map (fourth quarter-dependent variable)
30	**Growth and development (infrastructure)**	**Human resources**	Human resources quantity (Hu Res-Qua)	103	116	Lower-right area of the map (fourth quarter-dependent variable)
31			Employee behavioral factors (Emp-Be-Fac)	102	115	Lower-right area of the map (fourth quarter-dependent variable)
32			Quality of human resources training and education (Qua-Hu Res-Tra)	123	89	Upper-left area of the map (second quarter-drivers)
33		**Information system**	Research (Res)	101	114	Lower-right area of the map (fourth quarter-dependent variable)
34			Collection, processing, and analysis of information (Co-Pro-Ana-Inf)	105	94	Lower-left area of the map (third quarter-secondary leverage)
35			Information management structure (Inf-Man-Str)	102	91	Lower-left area of the map (third quarter-secondary leverage)
36		**Medicine and medical equipment**	Medical facilities and equipment in health system (Med-Fac-Equ-He)	124	124	Upper-right area of the map (first quarter-target variable)
37			Pharmaceutical system in health system	121	120	Upper-right area of the map (first quarter-target variable)
38	**Health financing**	**Resources collection**	Public resources (Pub-Res)	117	120	Upper-right area of the map (first quarter-target variable)
39			Government resources (Gov-Res)	121	118	Upper-right area of the map (first quarter-target variable)
40			Private resources (Pri-Re)	94	120	Lower-right area of the map (fourth quarter-dependent variable)
41			External resources (Ex-Res)	98	116	Lower-right area of the map (fourth quarter-dependent variable)
42			Factors affecting the amount of health financial resources (Fac-Amo-He-Fin-Res)	133	99	Upper-left area of the map (second quarter-drivers)
43		**Resources pooling**	Insurance mechanism (Ins-Mec)	135	118	Upper-right area of the map (first quarter-risk variable)
44		**Resources allocation and purchasing**	Resources allocation pattern (Res-All-Pat)	123	116	Upper-right area of the map (first quarter-target variable)
45			Medical services resources (Med-Ser-Res)	120	113	Upper-right area of the map (first quarter-risk variable)
46			Health services resources (He-Res-Ser)	121	115	Upper-right area of the map (first quarter-risk variable)
47			Service purchasing (Pur)	129	114	Upper-right area of the map (first quarter-risk variable)
48			Tariffs and pricing (Tar-Pri)	131	115	Upper-right area of the map (first quarter-risk variable)
49			Payment systems (Pay-Sys)	115	120	Upper-right area of the map (first quarter-target variable)
50	**Leadership & governance**	**Structural factors**	The power and role of political factors in the health sector (Pow-Rol-Pol-Fac)	125	116	Upper-right area of the map (first quarter-risk variable)
51			Roles, duties, and participation in practice (Rol-Dut-Par-Pra)	108	120	Lower-right area of the map (fourth quarter-dependent variable)
52			Inter-sectoral communication and cooperation (In-Sec-Co-Co)	107	115	Lower-right area of the map (fourth quarter-dependent variable)
53		**Develop laws and regulations**	Internal organizational laws (Int-Law)	96	120	Lower-right area of the map (fourth quarter-dependent variable)
54			Effective external laws (Ext-Law)	116	122	Upper-right area of the map (first quarter-target variable)
55		**Customer protection**	Support society / individuals health (Sup-He-Soc-Ind)	137	125	Upper-right area of the map (first quarter-target variable)
56			Financial support (Fin-Sup)	126	94	Upper-left area of the map (second quarter-drivers)
			Totals	6310	6310	

Figure 1 shows the position of the 54 key variables in the analysis of the indirect effects. Based on the analysis of the indirect effects, “environmental health,” “environmental and occupational risk factors,” and “ quality of human resources training and education “, “factors affecting the amount of health financial resources”, and “financial support” have more influence, are located in the upper left part of the map, and are the system’s drivers. Variables: “demographic factors related to population/patient”, “occupational health”, “biological risk factors”, “disease incidence”, “treatment measures/patient rights”, “improvement of health referral system”, “palliative and supportive care coverage”, “traditional medical care coverage”, “collection, processing, and analysis of information”, and “information management structure” have the most negligible impact and are considered as independent variables and Secondary levers in the system, among which, the variables “population lifestyle change”, “demographic factors related to population/patient”, “ collection, processing, and analysis of information”, and “information management structure” are considered as secondary levers and are of the most significant importance due to their proximity to the center and the horizontal axis.

Variables located in the upper right part of the map have the most importance and influence and dependency on the Iranian HS located above the bisector line, known as bidirectional or risk variables and the ones below it, known as target variables consisting of: “economic and social indicators”, “community empowerment”, “community care covered in family health, reproductive and sexual health, nutrition and food security”, “behavioral risk factors”, “inpatient services delivery”, “outpatient services delivery”, “medical facilities and equipment in HS”, “pharmaceutical system in HS”, “medical services resources”, “health services resources”, “public resources”, “government resources, “insurance mechanism”, “resources allocation pattern”, “tariffs and pricing”, “service purchasing”, “payment systems”, “the power and role of political factors in the HS”, “effective external laws”, and “ support society / individuals health”.

The variables “health indicators”, “mental health”, “rehabilitation care coverage”, “services efficiency”, “equity and access to services”, “services effectiveness”, “employee behavioral factors”, “human resources quantity”, “research”, “private resources”, “external resources”, “inter-sectoral communication and cooperation”, “roles, duties, and participation in practice”, “internal laws”, and “characteristics and capacity defined for each level of service” are located at the bottom right of the map and have a high dependence on the HS.

Therefore, the results of MicMac software and consideration of the drawing map identified the following nine variables as the driving variables and secondary levers. The order and importance of each are as follows based on the MicMac map:

1. Factors affecting the amount of health financial resources
2. Environmental and occupational risk factors
3. Financial support
4. Quality of human resources training and education
5. Environmental health
6. Population lifestyle change
7. Demographic factors related to population/patient
8. Collection, processing, and analysis of information
9. Information management structure

**Fig. 2:**
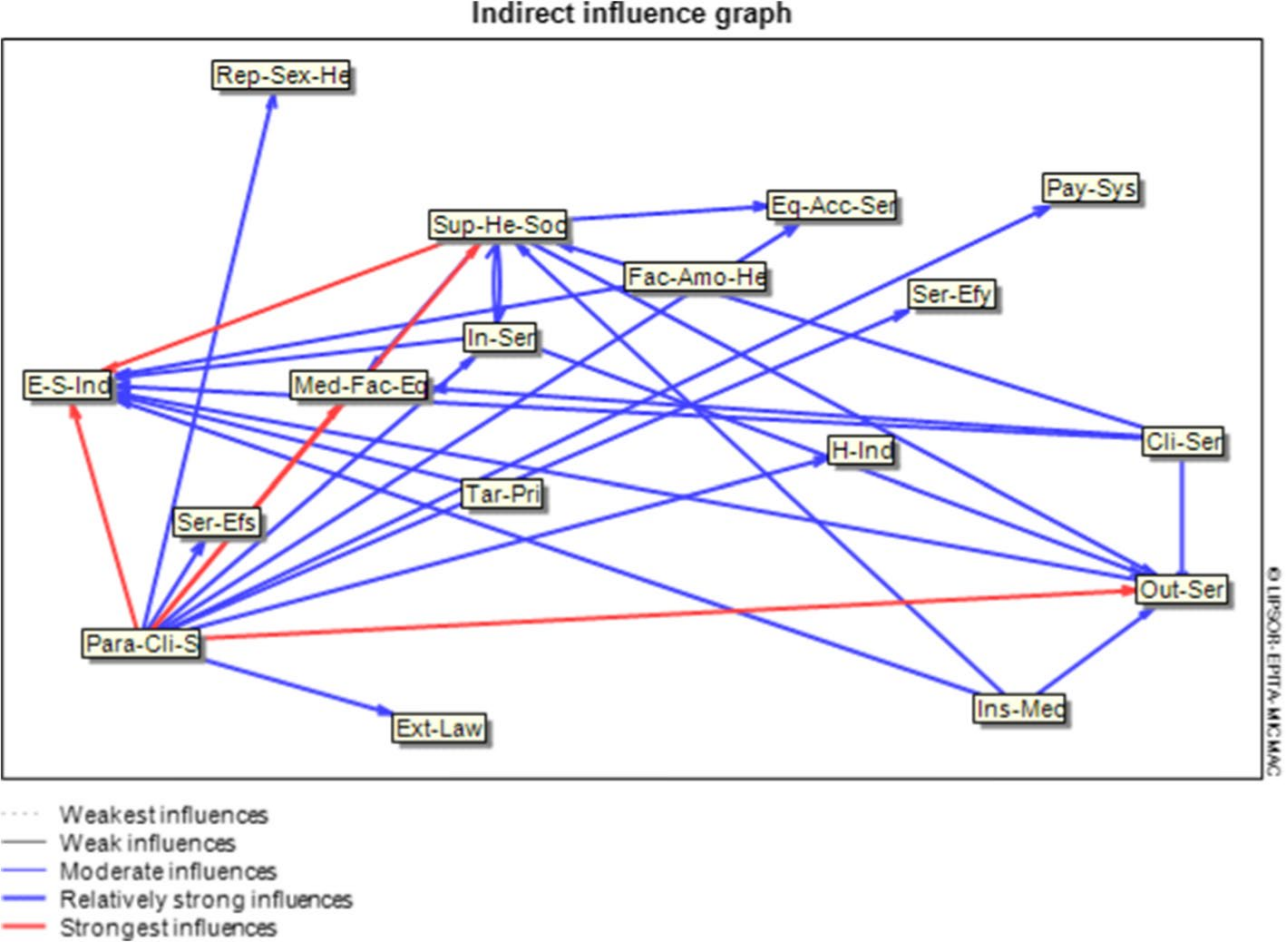
Results of analysis of indirect effects of target and risk variables in the Iranian HS

Figure 2 shows 1% of the vital target and risk variables. That should be considered more within the HS, with strong and relatively strong effective relationships based on indirect relationships. According to this figure, the provision of para-clinical and clinical services on variables such as economic and social indicators, outpatient services delivery, medical facilities and equipment, and support society / individuals health has a powerful and relatively strong effect, respectively. Para-clinical services have a fairly strong effect on variables such as health indicators, reproductive and sexual health, inpatient services delivery, services efficiency, equity and access to services, services effectiveness, payment systems, and effective external laws. The variables of inpatient services delivery and the insurance mechanism have a relatively strong effect on variables such as economic and social indicators, outpatient services delivery, and support society / individuals health. Variables of outpatient services, factors affecting the amount of health financial resources, and tariffs and pricing have a relatively strong relationship with economic and social indicators. Finally, support society / individuals health has a relatively strong effect on variables such as outpatient services delivery, inpatient services delivery, equity and access to services and medical facilities and equipment in HS. It has a strong relationship with economic and social indicators.

**Fig. 3:**
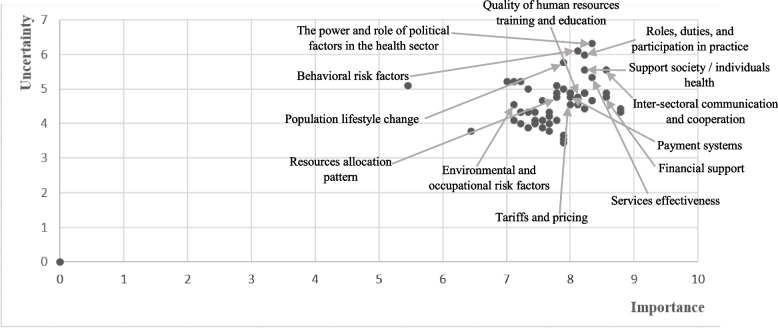
Extraction of health scenario-making factors based on the opinions of experts

The expert’s points of view were also analyzed, besides simultaneously analyzing the findings obtained from the software (Fig. 1). Accordingly, the components of “the power and role of political factors in the HS”, “high-risk behaviors and risk factors”, “roles, duties, and participation in practice”, “population lifestyle change”, “support society / individuals health”, “inter-sectoral communication and cooperation”, and “services effectiveness” had the most importance and uncertainty degree in the HS. In addition to the mentioned items, components such as “environmental and occupational risk factors”, “quality of human resources training and education”, “resources allocation pattern”, “tariffs and pricing”, “payment systems”, and “financial support” were also considered by the experts as the significant components (Fig. 3).

Finally, the following seven variables were selected as the scenario framework for Iran’s HS’s future based on MicMac software and experts’ importance-uncertainty scores.

1. Power, politics and communication network: governance
2. Lifestyle and behavioral factors: lifestyle
3. Quality of human resources training and education: human resources
4. Environmental and occupational risk factors: Environment
5. Payment and tariff system and allocation pattern: financing
6. Support society / individuals health
7. Services effectiveness: service delivery, especially para-clinical and outpatient services

Then a designed questionnaire was provided to experts in the form of a cross-impact-balance matrix (CIB). The next step was quantifying the qualitative expert views and summarizing the questionnaire results to provide the Scenario wizard input. The cross-impact-balance matrix is presented in Table 2.

**Table 2:**
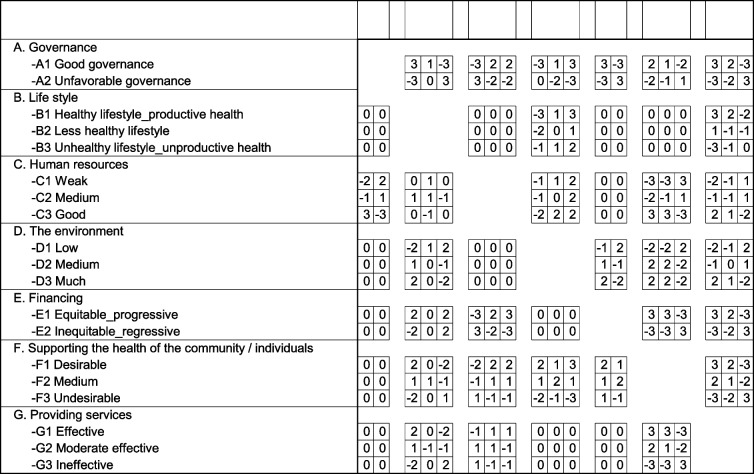
Cross-impact balance matrix of the main uncertainties of selected scenario-making factors in the Iranian HS.

Considering seven key uncertainties, five of which had three alternatives and two of which had two, 972 scenarios were constructed for the Iran’s HS that included all possible combinations. Logical scenarios with strong or relative compatibility were extracted through a working group meeting to identify interactions and then analyze the its results using the Scenario wizard. Since, based on the methodological recommendations, scenarios with a compatibility of 0 to about 2 can be used, finally, the general space of the six main scenarios resulting from the output of the Scenario wizard software was displayed in Table 3, and then the alternative scenario’s narration of Iran’s health system was developed. It is necessary to explain that in each scenario, the set of driving variables along with the possible alternative as well as existing relationship with each other are reported.

**Table 3:** Determining possible scenarios in the Iranian HS in terms of key uncertainties

Compatible scenario no. 6	Compatible scenario no. 5	Compatible scenario no. 4	Compatible scenario no. 3	Compatible scenario no. 2	Compatible scenario no. 1	
Unfavorable governance	Unfavorable governance	Good governance	Good governance	Good governance	Good governance	**Governance**
Less healthy lifestyle	Unhealthy lifestyle: unproductive health	Healthy lifestyle: productive health	Healthy lifestyle: productive health	Healthy lifestyle: productive health	Healthy lifestyle: productive health	**Life style**
Weak	Weak	Medium	Good	Medium	Good	**Manpower**
Much	Medium	Much	Much	Much	Much	**The environment**
Inequitable: regressive	Inequitable: regressive	Equitable: progressive	Equitable: progressive	Equitable: progressive	Equitable: progressive	**Financing**
Undesirable	Undesirable	Medium	Medium	Desirable	Desirable	**Support society / individuals health**
Ineffective	Ineffective	Effective	Effective	Effective	Effective	**Providing services**
37	36	56	62	64	69	**Total Impact Score**
0	1	−2	-1	-1	1	**Consistency Value**

In scenario number one, called the desired scenario, a set of most favorable uncertainties have been realized. In this scenario, social equity is the most critical perspective of the HS. Accordingly, the governance applied by government agents in the HS will function as desired. Good governance will have a significant impact on other functions of the HS and help in creating a system for better management by designing an appropriate organizational structure to achieve organizational goals; formulating policies and regulations and coherence between them; being accountable and responsive with flexibility; proper selecting and appointing individuals and public participation in the policymaking process; networking and establishing open and transparent communication on HS priorities and effective inter-sectoral cooperation.

Therefore, the number of human resources in the health sector will be determined based on the covered population. They will receive adequate and appropriate training during their starting and during-service time. Increasing motivation and satisfaction and reducing organizational corruption are essential issues in human resources management.

Good governance will necessitate the HS to respond to patients’ demands and preferences and design appropriate care packages. In addition, setting a proper referral system and leveling services will increase the service’s efficiency and effectiveness and reduce health sector costs. Also, vital monitoring and right role-playing have prevented induced demand, especially in para-clinical and outpatient services.

The number of hospital beds is proportional to the population, updated medical technologies provide health services, and the medicine is sold based on the population’s needs in medical centers and pharmacies in this scenario.

Financing relies more on the insurance system to put a less financial burden on individuals/patients, and payment will be based on the individual’s affordability. On the provider’s side, payment will be made based on their performance to deliver qualified services and align the HS’s activities with the system’s predetermined goals.

Health Advocacy is another goal of the government that tries to reform the community policies in line with health plans by defining strategies, plans, and activities. Improving lifestyle psychologically and physically, avoiding high-risk behaviors (such as inactive lifestyle, obesity, smoking and alcohol consumption, chronic diseases, and psychosocial stress), and environmental and occupational risk factors are issues that require other economic and social sectors’ broad support in this scenario. Therefore, there is a healthy lifestyle in society and simultaneouslys, attention to environmental and occupational risk factors has reduced the disease incidence in the population through the MoHE’s support, appropriate information technologies, and community accompaniment.

As a result, this scenario will improve the health, economic, and social indicators that will provide equal access to health services to all members of the society, making it the most optimistic scenario of the study.

Scenarios number two, three, and four are also the desirable scenarios of the study like scenario one; however, considering the issues like human resources training, competence, and skill, their motivation and job satisfaction are considered Intermediate in scenarios number two and four and design of appropriate patient care packages and attention to patients’ demands and preferences are considered moderate in scenarios number three and four.

Scenario number five, called the disaster scenario, which is the most pessimistic compatible scenario, unfavorable governance dominates the HS. In other words, health and equity are not essential either in society or in the individual dimension. In achieving the HS goal, health and access to services, the HS governance has not been able to formulate appropriate policies and regulations, and as a result, the strategic orientation of the system is not well defined. At the executive level, skilled and competent human resources are not employed, and in some areas, the system faces a human resources shortage. As a result, meeting the community’s needs and access to health services will not be possible.

The referral system and service delivery levels don’t work well in this scenario, which will exacerbate the above problems such as inefficiency and ineffectiveness of services, increase the financial burden, and reduce the economic growth.

Job satisfaction decreases in this scenario due to inefficient payment systems, consequently increasing induced demands, informal payments, and health corruption. It will prevent providing effective services and impose a heavy financial burden on both the provider side due to inefficiency and the demand side.

Increased demand, shortage of diagnostic and treatment beds, low drug capacity, or minority access of the population due to their low financial capacity are common problems of the HS. In addition, new medical technologies in laboratory diagnostic and imaging services raise the induced demand for para-clinical and outpatient services and turn the HS system into a market.

Insurance funds are not integrated into HS financing, which imposes increasing costs in fund management, and also risk pooling and cross-subsidies are not done well and Individuals/patients will pay their expenses to the HS according to their needs, leading to increased catastrophic costs and reduced the poor people’s access to health services; in other words, it will make the activities not aligned with patients’ needs and preferences.

People’s lifestyle is unhealthy, and high-risk behaviors such as drug addiction, alcohol consumption, obesity, chronic diseases, and inactivity are common in this scenario. The government could not attract the support of other related social sectors, leading to an increase in the disease burden and the services demand. Nevertheless, environmental and occupational risk factors, both at the governmental and executive levels, have been moderately considered, and the necessary advocacy has been provided in this area.

All the mentioned issues in this scenario lead to the HS weakness, which increases the burden of disease and growing demand for services, imposes a heavy economic burden on both society and the government, and as a result, increases Poverty, violence, corruption, declines health, economic and social indicators, and limits equal access to health services. These consequences exacerbate the HS’s growing problems and shake government macroeconomics.

In Scenario number six, health and equity in the individual dimension are the most critical perspective of the HS. Service delivery components are ineffective, financing is unfair, and community health support and governance are undesirable at the macro-level. Although governance is not desirable, it has a moderate performance in applying effective interventions on health and attracting the other sector’s support and cooperation. There is much attention to environmental and occupational risk factors and less healthy lifestyles, with moderate attention to behavioral risk factors that the burden of disease and mortality is reduced, health is promoted, and the services demand is reduced. Other components have also malfunctioned due to the weakness in management and planning issues at the macro and micro levels of the HS, weak monitoring of implementation, and weak organizational culture and the indicators of equity/access to services, effectiveness and efficiency of the system are not in a good state at the community level.

Not employing talented, competent, and need-based human resources with unfair payment systems, like the fee for services system, has led to ineffective services provided to society that increase the induced demand and impose a financial burden in some cases. Unlike Scenario Number 5, the community and other relevant sectors pay more attention to health-related issues, the risk factors and risky behaviors. Therefore, the economic and social situation of the government and consequently health, economic and social indicators is better than the previous scenario and is preferable to scenario number 5.

## Discussion

### Summary of key findings.

Despite the complexity of the relations in the HS, a sectoral look at the system with paying too much attention to details lead to ambiguities and challenges in designing and implementing policies and achieving the HS’s final goal. While many similar studies in the HS have evaluated and measured the factors affecting each dimension of the HS [12–14], the current study has tried to present tangible images and different and believable stories of probable and compatible scenarios of the Iran HS’s future, while considering all dimensions and functions, such as financing, resources provision, providing services and stewardship, as well as considering the necessary features to adopt a future-oriented approach, including the complexity, ambiguity, uncertainty and the need for holism.

This study has identified seven scenario-development components for the HS’s future as input for Scenario Wizard. These variables, especially governance, human resources training, and financing, have a high degree of uncertainty and instability based on the Mick Mac diagram, which has caused the Iranian HS to be unsustainable in general.

So that Doshmangir et al. in 2019 [20], Khankeh et al. in 2021 [21], and Dehnavieh et al. in 2019 [22] emphasized the instability of financial resources, political and economic instability, and managerial and economic instability in Iran’s health system, respectively and they have stated that these issues will always prevent the promotion of fair care and the provision of effective and efficient services.

Six images of the future of the Iranian HS or alternative paths to it were described and narrated in the form of six main scenarios. The first to fourth scenarios are desired from experts’ points of view and can be the basis for context-based policy-making and planning. In fact, the fifth scenario consists of a set of the most unfavorable conditions that provide a horrible picture of the future. The sixth scenario has been evaluated as undesired and contains tips, such as reminders and avoidance, when making relevant health-related decisions.

In 2021, Esmaeili et al. conducted a study entitled designing scenarios of the health system at the national level. The results of this study are relatively consistent with the results of the present study. Therefore, they explained four scenarios. In scenario number one, a suitable diet model has been ruling the society due to the proper structure of the country’s HS, the participation of institutions and organizations, the integration of insurances, the development of information technology and the cooperation of people and its consequence has been to save the costs of medical care and maintain and improve the health of citizens. In scenario number two, equity in the collective dimension and in scenario number three, welfare in the individual dimension are the most important perspectives of the country’s HS and in scenario number 4, called the disaster scenario, there is neither equity in the collective dimension nor welfare in the individual dimension [23].

### Guideline recommendations for moving the HS towards a better future

The first important principle in improving the unfavorable scenarios is good governance in the HS so that it can have its effects and consequences in infrastructure issues including financial, human, and information related issues; visioning; laws, and regulations affecting health promotion and access to the required health services for all the community members; asserting authority at the political and executive levels; and cooperating with other economic and social sectors affecting the health.

In line with the present study and due to the impact of governance on all dimensions of the system, Mehrollhassani et al. conducted a study entitled “providing a comprehensive conceptual framework for futures studies in health” in 2019, using a critical method of futures studies and the causal layered analysis (CLA). They have considered the doctrine and philosophy of society and the discourse of governance in the deep and discourse layer of society [24].

Jafari et al. argue that governance in the HS is related to laws, regulations, and policies in health promotion and is a tool that society uses to ensure conditions in general. It enables citizens to live with the highest level of health and well-being. Therefore, designing a customized model of good governance is essential in different societies’ HSs, and dimensions such as legislation, transparency, responsibility, equity, efficiency and effectiveness, accountability, and consensus should be considered in the analysis of good governance of each country [25]. For example, developing an integrated model including requirements and objectives; merger of some vices and departments of the MoHE; developing more connections with relevant organizations outside the MoHE; determining guiding principles for the formulation of health sector laws and regulations; more use of research evidence in health policy and planning; strengthening the HS leadership, management and stewardship and developing comprehensive quality standards for the evaluation and accreditation of health services are suggested for the governance of Iran’s health system [26].

The second principle is sustainable financial resources and effective management of resources to achieve efficiency, effectiveness, and equal access to health services. According to this principle, service packages are defined based on the needs and preferences of individuals, and the tariff and pricing system functions based on the individual’s income and the economic and social situation of the country while providing services appropriate to each level of service delivery to people in need. With the increasing role of insurance in financing services, the demands of society will be responded to more appropriately. It is better to minimize the negative impact of annual inflation, which affects the amount of invested financial resources in each system, on vital systems such as health.

Hashemkhani Zolfani et al. also state that according to the standards and principles of sustainability, health financing should be considered in any economic, social, and environmental fluctuations like changes in oil prices, other possible sanctions, population aging, etc., in their study on the future of Iran’s health financing in 2019 [27].

Mossadegh Rad et al. in 2021 [28] and Sepetis [29] in 2020 emphasize the sustainability of the financial resources in health and state that policymakers and senior managers should take action to strengthen the sustainability of the health financing system. Accordingly, the most frequent strategies to have sustainable financing in the HS are increasing the share of health in GDP, expanding tax revenues, using advanced health payment methods, strengthening public-private partnerships, increasing the efficiency of the HS, reducing HS costs, consolidating insurance funds, eliminating insurance overlap, determining support packages based on health needs, tariffs based on health services value and applying fixed and performance-based payment methods to health service providers [28].

Meng et al. Emphasize that a significant increase in insurance coverage and reimbursement of hospital costs will lead to a rise in the use and coverage of health care. In addition, with access to primary health care coverage, risk protection will be more robust with more efficiency and qualified care [30].

The third principle is required human resources in terms of quantity and quality. Since training the required human resources is one of the main functions of the HS, so employing skilled, competent, and knowledgeable forces will improve the HS performance and the community health subsequently. Therefore, it is recommended to cultivate and institutionalize the essential values of the system at the micro and macro levels and use motivational tools to achieve this principle.

In their study in 2016, Sefiddashti et al. state that the proper distribution of human resources in the HS is very important to improve people’s health. Therefore, it is necessary to have information about the level of equality in distribution of health human resources and their time trends for better planning and optimal use of these resources [31].

In one of the futures studies of Iran hospitals in 2016, it was mentioned that improving training and in-service training improve the organization’s efficiency and flourish the talents, especially in hospitals. This study emphasizes creativity and innovation in hospitals and states that they are necessary for all organizations’ survival and enhancing team working is essential according to conditions [32].

In its 2013 study, Asuquo states that improving the HS requires effective management and leadership skills, holistic and flexible thinking, and twenty-first century conceptual, human, and technical skills. More importantly, effective communication and motivational skills are vital in leading and creating capable and cohesive teams [33].

Applying these three principles in policy-making and planning according to the context and requirements of the country, can significantly improve the status of the study variables and other components affecting health and the future of the Iran HS. As a result, it will provide ground for access to health services, health literacy, and effective and efficient services for all the community.

#### Conclusions

This research has faced challenges like bringing a diverse range of experts together in all relevant fields. However, based on the findings of this study, key components were identified, and by structural analysis of the relationships between them in Mick Mac software, the variables “power and politics and communication network”, “lifestyle and Behavioral factors”, “quality of human resources training and education”, “Environmental and occupational risk factors”, “Payment and tariff system and allocation pattern”, “support society / individuals health “, and “services effectiveness “ as drivers. Then six future scenarios of Iran’s HS were mapped and narrated, extracted from possible and compatible alternative futures based on the results of Scenario wizard software, which is a reasoned and reliable basis for designing any strategy and policy in the field.

Effective interaction and constructive interventions at the policy-making and execution level of the HS in Iran require continuous monitoring of critical components identified in this study in each of the seven main trends. Given that the Iranian HS is not stable and has many complexities, the development of management and planning science based on the context and economic conditions of the country and strengthening advocacy in the HS (good governance), stable and logical financial resources, and paying attention to the necessary infrastructure in this area and training qualified and required human resources in terms of quantity and quality, are the underlying factors to guide internal trends towards images of more desired scenarios.

## Data Availability

All data generated or analyzed during this study are included in this published article.
